# Is “Sexual Competence” at First Heterosexual Intercourse Associated With Subsequent Sexual Health Status?

**DOI:** 10.1080/00224499.2015.1134424

**Published:** 2016-02-18

**Authors:** Melissa J. Palmer, Lynda Clarke, George B. Ploubidis, Catherine H. Mercer, Lorna J. Gibson, Anne M. Johnson, Andrew J. Copas, Kaye Wellings

**Affiliations:** ^a^ Department of Population Health, London School of Hygiene and Tropical Medicine; ^b^ Department of Social Science, Institute of Education, University College London; ^c^ Research Department of Infection and Population Health, University College London; ^d^ Department of Social and Environmental Health Research, London School of Hygiene and Tropical Medicine

## Abstract

The timing of first sexual intercourse is often defined in terms of chronological age, with particular focus on “early” first sex. Arguments can be made for a more nuanced concept of readiness and appropriateness of timing of first intercourse. Using data from the third National Survey of Sexual Attitudes and Lifestyles (Natsal-3), conducted in 2010–2012, this study examined whether a context-based measure of first intercourse—termed *sexual competence*—was associated with subsequent sexual health in a population-based sample of 17-to 24-year-olds residing in Britain (*n* = 2,784). Participants were classified as “sexually competent” at first intercourse if they reported the following four criteria: contraceptive protection, autonomy of decision (not due to external influences), that both partners were “equally willing,” and that it happened at the “right time.” A lack of sexual competence at first intercourse was independently associated with testing positive for human papillomavirus (HPV) at interview; low sexual function in the past year; and among women only, reported sexually transmitted infection (STI) diagnosis ever; unplanned pregnancy in the past year; and having ever experienced nonvolitional sex. These findings provide empirical support for defining the nature of first intercourse with reference to contextual aspects of the experience, as opposed to a sole focus on chronological age at occurrence.

The timing of first sexual intercourse has long been of public health concern, but the way in which acceptability is defined has changed over time. Where once the focus was on occurrence before marriage (Hitchens & James, 1965; Reiss, 1965), more recently it has shifted to chronological age, with “early” sex typically being described as occurring before the legal age of sexual consent, for example, or the age of majority. The case for a more nuanced measure of timing of sexual debut has been made on the grounds that the use of chronological age neglects individual differences in physical, social, and psychological maturity, and also cultural variation in social norms and legislation governing timing of sexual initiation (Hawes, Wellings & Stephenson, 2010). In recognition of this, an attempt was made in the second British National Survey of Sexual Attitudes and Lifestyles (Natsal-2) to broaden the criteria by which the appropriateness of timing of onset of sexual activity is measured (Wellings et al., 2001) and to bring them more in line with the broad definition of sexual health endorsed by the World Health Organization (WHO, 2006). On a priori grounds that first intercourse should be safe, consensual, an autonomous decision, and optimally timed, a combined variable was constructed using answers to four corresponding survey questions and given the working label of *sexual competence*. This study examined whether this measure of sexual competence at first intercourse is associated with subsequent sexual health.

## Age

Chronological age is a primary social and cultural category and is often used in research. However, it has been argued that age itself is an empty variable, given that one rarely assumes age per se causes a behavior (Settersten & Mayer, 1997). It is whatever attributes age is a proxy for that is thought to be important, for example, physical development and emotional maturity (Schwall, Hedge, & Borman, 2012). With reference to Neugarten and Hagestad (1976), Settersten & Mayer (1997) argued that “chronological age is often a poor indicator of biological, social, or psychological age” (p. 240) because there are significant individual differences in development. In the context of public health, the timing of first sex tends to be considered in terms of its relevance in predicting adverse sexual health outcomes—and is commonly operationalized as a dichotomous variable to define “early” first intercourse. The threshold for what is classified as early sex in the literature often reflects the legal age of consent in the country under study (age 16 in Britain). However, the age of consent is a somewhat arbitrary threshold, varying considerably over time and across cultures (Waites, 2005), and cross-national studies have shown only a weak correlation between the legal age of consent and the age seen as appropriate for sexual initiation (Madkour et al., 2014). Early sexual activity has been found to be associated with increased risk of acquiring sexually transmitted infections (STIs) (Eberhart-Phillips et al., 2001; Kaestle, Halpern, Miller, & Ford, 2005; Kahn, Rosenthal, Succop, Ho, & Burk, 2002), motherhood and abortion before age 18 (Wellings et al., 2001), and unplanned pregnancy (Wellings et al., 2013). It is this predictive ability of “early” first intercourse which is drawn upon as justification for studies focusing on this as a negative public health outcome.

## Context of First Intercourse

To date, few attempts have been made to interpret timing of first intercourse in terms of attributes other than age (Hawes et al., 2010). The most common contextual factor of first intercourse given consideration in research tends to be contraceptive use, and studies have examined the extent to which this is predictive of subsequent use. For example, using data collected from female students in Britain, Parkes, Wight, Henderson, Stephenson, and Strange (2009) found that the contraceptive method used at first intercourse was predictive of using a similar method at most recent intercourse, and those who used no effective method that first time were subsequently at increased pregnancy risk. Similarly, analyses of Slovenian and U.S. data have found evidence of a strong association between condom use at first intercourse and use at subsequent sexual encounters (Klavs, Rodrigues, Wellings, Weiss, & Hayes, 2005; Shafii, Stovel, & Holmes, 2007; Stulhofer, Bacak, Ajdukovic, & Graham, 2010).

There has been relatively less research on the association between the social and emotional aspects of first sex and their implications for subsequent sexual health and wellbeing (Hawes et al., 2010). Using data from the US National Health and Social Life Study (NHSLS), Else-Quest, Hyde, and DeLamater (2005) classified their participants as having had their first sexual experience in a negative context if any of the following criteria applied: first intercourse was forced, was with a blood relative, or was with someone who paid the participant to have sex; the main reason the participant chose to have first intercourse was peer pressure or the influence of drugs or alcohol; or the participant reported having been touched sexually by an adult prior to puberty. In addition, female respondents were also classified as having had a negative first sexual experience if first intercourse occurred with a stranger, someone they had just met, or with someone who they did not know well. Analyses found that a negative context of first sexual experience was associated with sexual dysfunction, more sex guilt, poorer general health, experience of STIs, and poorer life satisfaction. Other studies have demonstrated an association between more negative emotional reactions to first intercourse, and subsequent sexual functioning and satisfaction (Reissing, Andruff, & Wentland, 2012; Smith & Shaffer, 2013).

## Sexual Competence

The assessment of “sexual competence” at first sexual intercourse aims to increase the sophistication with which the concept of timing is treated empirically. This approach takes the view that sexual activity among young people is not inherently negative or risky, and that a consideration of the appropriateness of sexual activity based solely on chronological age neglects the importance of contextual factors in defining the quality and safety of a sexual encounter. Such an approach is consistent with the substantial body of literature within the field of adolescent sexuality that calls for sexuality to be recognized as a normative aspect of young persons’ development (Allen, 2007; Diamond, 2006; Ehrhardt, 1996; Halpern, 2010; Tolman & McClelland, 2011; Tolman, Striepe, & Harmon, 2003). Sexual activity during adolescence has traditionally been framed as a “problem behavior,” often listed alongside smoking, alcohol use, and drug use as a cluster of behaviors regarded as causes for concern (Donovan & Jessor, 1985). However, Halpern (2010) argued that “adolescent sexual behaviour is distinct from other problem behaviour because its undesirability is primarily a function of age and assumed immaturity, rather than intrinsic and inevitable health risk” (p. 6). An approach that emphasizes the contextual factors in defining the nature of first sexual intercourse may also be more compatible with the priorities of young people; qualitative research has found that young people tend to consider the legitimacy of engaging in sexual encounters to be better located in the notion of “readiness” as opposed to age-defined legality (Thomson, 2004).

The concept of “competence” was first used in the context of sexual initiation by Wellings et al. (2001), extending its previous application to sexual lifestyles in work on interactional competence between sexual partners (Ingham & Van Zessen, 1997; Vanwesenbeeck, Van Zessen, Ingham, Jaramazovic, & Stevens, 1999). Sexual competence at first sexual intercourse was operationalized using four criteria that were captured by self-reported variables measured in the second National Survey of Sexual Attitudes and Lifestyles (Natsal-2): contraceptive protection, self-perceived consensuality (equal willingness of both partners), self-perceived autonomy (not due to external influences such as alcohol or peer pressure), and self-perceived acceptable timing (i.e., that it occurred at the “right time”). (The latest Natsal survey questions, asked in the Natsal-3, are detailed in Table 1.) Participants were classified as sexually competent at first intercourse if they reported that the event met each of the four conditions.

**Table 1.** Natsal-3 questions used to construct measure of ‘sexual competence’ at first intercourse. Highlighted are the answers that must be given for a respondent to be classified as ‘sexually competent’ at first intercourse

Concept	Natsal-3 Questions	Coding
**Willingness of partners**	**Q**: Would you say you were both equally willing to have intercourse that first time, or was one of you more willing than the other?	Equally willing = 1 if answer = 1
	**A**:	
	1. Both equally willing	Equally willing = 0 if answer = 2 or 3
	2. Respondent more willing	
	3. Partner more willing	
**Acceptable timing**	**Q**: Looking back now to the first time you had sexual intercourse, do you think….	Right time = 1 if answer = 3
	**A**:	
	1. You should have waited longer before having sex with anyone	Right time = 0 if answer = 1 or 2
	2. That you shouldn’t have waited so long	
	3. It was at about the right time	
**Autonomous reason**	**Q**: Which one of these applied to you at the time…. (choose the main one that applied at the time)	Autonomous reason = 1 if answer =1 or 2 or 4 or 8 or 9
	**A**:	Autonomous reason = 0 if answer = 3 or 5 or 6 or 7
	1. I was curious about what it would be like	
	2. I was carried away by my feelings	
	3. Most people in my age group seemed to be doing it	
	4. It seemed like a natural ‘follow on’ in the relationship	
	5. I was a bit drunk at the time	
	6. I had smoked some cannabis	
	7. I had taken some other drugs	
	8. I wanted to lose my virginity	
	9. I was in love	
	10. Can’t choose/more than one main factor	
**Use of reliable method of contraception**	**Q**: Thinking of that first time you had sexual intercourse, did you or your partner use any form of contraception or take any precautions that first time, or not?	Reliable contraceptive protection = 1 if answer = 1 or 2
	**A**:	Reliable contraceptive protection = 0 if answer = 3 or 4 or 5 or 6 or 7 or 8
	1. Condom	
	2. The pill	
	3. Emergency contraception	
	4. Other contraception	
	5. (Partner) withdrew	
	6. Made sure it was a safe period	
	7. No precautions by me, don’t know about partner	
	8. No precautions by either of us	

We note the potentially negative evaluative connotations of the term *competence*; however, it is applied in this study for consistency with previous publications using Natsal data. Nonetheless, the term should be considered provisional.

The use of these four domains in defining sexual competence sought to reflect the definition of sexual health endorsed by WHO (2006), which stresses the importance of not only the biomedical aspects of sexual health but also mental and social well-being, referring to a “positive and respectful approach to … sexual relationships” and “safe sexual experiences, free of coercion.” The measure of sexual competence at sexual debut aims to provide a more comprehensive account of the nature of the first sexual encounter, including not only protection against negative physical sexual health outcomes but also the more emotional and social aspects concerned with willingness and feelings that follow the event. The WHO definition of sexual health relies on multiple factors, all of which should be fulfilled for sexual health, as opposed to any “either/or” or hierarchical relationships of the different typologies of health. Thus, the measure of sexual competence was operationalized dichotomously, such that each of the four components must be positively endorsed to be classified as sexually competent at first intercourse.

The questions used to construct the Natsal measure of sexual competence in this study are asked specifically about first heterosexual *intercourse*. This focus on penetrative, penile-vaginal intercourse is common in the sexual health literature; however, it obscures the fact that young people often engage in a wider array of noncoital activities prior to having sexual intercourse for the first time (Halpern-Felsher, Cornell, Kropp, & Tschann, 2005; O’Sullivan & Brooks-Gunn, 2005; Schwartz, 1999). Nonetheless, qualitative research has suggested that penetrative intercourse is commonly regarded by young heterosexuals as what constitutes “proper sex,” during which manhood is “achieved” and virginity “lost” (Bersamin et al., 2007; Holland, Ramazanoglu, Sharpe, & Thomson, 2000/2010), with precoital activities considered to be “building-up to” intercourse (Lewis, Marston, & Wellings, 2013). Therefore, a possible interpretation is that first intercourse constitutes a particularly salient first in young people’s sexual trajectories. Unless otherwise specified, where this article refers to *first sex* and *sexual debut*, we are concerned specifically with the first occasion of penetrative sexual intercourse.

## Aims of This Study

The measure of sexual competence at first intercourse has been used in a number of analyses of data from the Natsal studies (Mercer et al., 2005; Mitchell et al., 2013; Wellings et al., 2013; Wellings et al., 2001), has informed the construction of equivalent or similar measures used in other studies (Heron et al., 2015; Testa, Coleman, & Trust for the Study of Adolescence & Naz Project London, 2006); and features on the WHO list of recommended indicators for monitoring progress toward the Millennium Development Goal of universal access to sexual and reproductive health (WHO and United Nations Population Fund [UNFPA], 2010). However, there has been no dedicated examination into whether this measure is predictive of subsequent sexual health. In this study, analysis of data from the third and most recent Natsal survey was used to examine whether sexual competence at first sexual intercourse is associated with subsequent sexual health status in a population-based sample of 17- to 24-year-olds in Britain. Specifically, the primary aim was to assess whether young people who were classified as not sexually competent at first intercourse were at greater risk of poor sexual health as indicated by experience of STIs, low sexual function, unplanned pregnancy, and nonvolitional sex. We were also interested in how the measure of sexual competence compares with the widely used indicator of “early” first intercourse (defined as that which occurs before age 16, the age of consent in Britain), in terms of the two measures’ associations with subsequent sexual health.

## Method

### Participants

The Natsal-3 is a stratified probability sample survey of 15,162 men and women aged 16 to 74 residing in Britain. The sampling strategy included a “boost” of 16- to 34-year-olds, resulting in data being collected from a total of 3,689 participants aged 16 to 24 at interview (Erens, Phelps, & Clifton, 2013). The Natsal-3 study was approved by the Oxfordshire Research Ethics Committee A (reference: 09/H0604/27). Participants provided oral informed consent for interviews.

### Data Collection

Participants were interviewed in 2010 through 2012 using a combination of face-to-face, computer-assisted personal interviews and computer-assisted self-interviews. Participants were asked about their age at, and experience of, first heterosexual intercourse using show cards in the face-to-face component of the interview. (The questionnaire used is available at http://www.natsal.ac.uk).

### The Measure of “Sexual Competence”

The binary variable *sexual competence* at first intercourse was constructed as described in Table 1 in accordance with its use in previous publications (Mercer et al., 2005; Mitchell et al., 2013; Wellings et al., 2013; Wellings et al., 2001). Participants were categorized as sexually competent at first intercourse if they reported the event was characterized by each of the following conditions: consensuality, acceptable timing, autonomy of decision, and contraceptive protection. Those who met fewer than all four conditions were categorized as not sexually competent at first intercourse. To meet the criteria of consensuality and acceptable timing, participants had to report that they and their partners were “both equally willing” to have intercourse that first time; and that, looking back, they thought it happened “at about the right time,” respectively. The question used to derive the autonomy of decision component consisted of nine answer categories, which were coded to distinguish between those that could be considered as external to the self (influence of alcohol, drugs, or perception of peers having sex) and those more representative of internal motivations (such as curiosity). It should be noted that this criterion does not seek to imply what are “good” or “bad” reasons to have sex for the first time, but rather, as the label autonomy suggests, reasons that are intrinsic, as opposed to extrinsic, to the self. Finally, the use of a condom and/or contraceptive pill was coded as having used a reliable method of contraception. This means participants who used only the pill would not have been protected against STIs; however, in this sample, of those coded as being “protected,” over 90% of reported using a condom. Therefore, condoms are the major contraceptive method contributing to these analyses. Confirmatory factor analysis of this measure have demonstrated that these four components, as currently coded, are significantly and positively correlated with one another and load on to a single common factor (Palmer, 2015a).

The survey questions used to construct the measure of sexual competence were not asked about first same-sex sexual experiences, so for the purpose of this article, we consider only first heterosexual intercourse. Where participants reported that they first had heterosexual intercourse at 12 years old or younger, the questions related to the circumstances and experience of first sex were asked about their first sexual encounter since turning 13. This was done with the aim of avoiding probing questions about sexual encounters in childhood which may have been nonconsensual (Erens et al., 2013).

### Measures of Subsequent Sexual Health

The selection of indicators of subsequent sexual health status (the outcome measures in this study) was informed by the multifaceted definition of sexual health endorsed by the WHO (2006), and recent calls in the literature to consider sexual health in its broader sense (Wellings & Johnson, 2013). Measures of STI acquisition and unplanned pregnancy were used as biomedical indicators of sexual health, while measures of sexual function and nonvolitional sex were used as psychosocial indicators.

For the STI outcomes, two measures were used: one based on answers to the question “Have you ever been told by a doctor or other healthcare professional that you had any of the following?” followed by a list of STIs (see Table 2 for STIs). The second was based on urine tests conducted on a subsample of the Natsal-3 sample (*n* = 1,597 of sexually active 17- to 24-year-olds) for several STIs, including chlamydia, gonorrhea, human papillomavirus (HPV), and human immunodeficiency virus (HIV), which was linked to the survey data by participant identification number (Sonnenberg et al., 2013). Due to the limited number of participants testing positive for bacterial STIs, only the results of HPV testing were used as an outcome measure in this study. An in-house Luminex-based genotyping assay (Bissett et al., 2011) was used to detect high-risk HPV (types 16, 18, 31, 33, 35, 39, 45, 51, 52, 56, 58, 59), indeterminate/possible high-risk HPV (types 26, 53, 66, 70, 73, 82), and low-risk HPV (types 6, 11).

**Table 2.** Percentage (95% CI) of sexually active 17-24 year olds reporting/experiencing key sexual health indicators, by gender^b^

	Women			Men		
	%	95% CI	N (non-weighted/weighted)	%	95% CI	N (non-weighted/weighted)
**Ever STI diagnosis^e^**	19.8	17.6, 22.3	1554/873	8.9	7.3, 10.9	1217/904
**Urine test HPV positive at interview^c^**	46.0	41.3, 49.8	863/521	16.6	13.8, 19.9	711/532
**Low sexual function in last year**	13.3	11.5, 15.5	1514/847	14.4	12.4, 16.8	1162/864
**Unplanned pregnancy in last year^d^**	2.8	2.1, 3.9	1485/837	.	.	.
**Ever experienced non-volitional sex^f^**	7.0	5.7, 8.5	1493/839	.	.	.

^b^Denominator is participants aged 17-24 at interview, who reported first heterosexual intercourse aged 13+. Participants reporting that they were ‘forced’ at first intercourse excluded (n = 22).

^c^‘Low’ risk and ‘high’ risk HPV. Denominator: all those who had a test result for HPV from urine sample.

^d^Pregnancies with unknown outcome excluded (n = 61).

^e^Survey question “Have you ever been told by a doctor or other healthcare professional that you had any of the following? Chlamydia; Gonorrhoea; Genital Warts (venereal warts); Syphilis; Trichomonas vaginalis (Trich, TV); Herpes (genital herpes); Pubic lice / crabs; Hepatitis B; (Men only:) NSU (Non Specific Urethritis), NGU (Non Gonococcal Urethritis); (Men only:) Epididymitis; (Women only:) Pelvic Inflammatory Disease (PID, salpingitis); (Women only:) Bacterial vaginosis; Yes, but can't remember which; None of these”

^f^Those who report occurrence of non-volitional sex at an age younger than first consensual intercourse excluded (n = 20).

Sexual function was measured using a psychometrically validated measure specifically developed for use in community surveys: the Natsal-SF (Mitchell, Ploubidis, Datta, & Wellings, 2012). The 17-item instrument includes questions on experience of specific sexual problems and level of general satisfaction and distress with sex life in the year prior to interview. Sexual function scores for each participant were derived, with low sexual function being categorized as a score within the lowest quintile (Mitchell et al., 2013).

Unplanned pregnancy was measured using the six-item psychometrically validated London Measure of Unplanned Pregnancy (LMUP) (Barrett, Smith, & Wellings, 2004). Women were asked six questions about pregnancies occurring in the year prior to interview, the responses to which were scored and summed on a scale of 0 to 12, with scores of 0 to 3 classified as an unplanned pregnancy (Wellings et al., 2013).

To measure the outcome of subsequent nonvolitional sex, participants were asked, “Since the age of 13, has anyone tried to make you have sex with them, against your will?” Those who answered yes to this were asked a follow-up question: “And since the age of 13, has anyone actually made you have sex with them, against your will?” Participants who answered yes to this second question were coded as having experienced nonvolitional sex. Heterosexual sex was defined for participants as including “vaginal, oral, or anal,” and same-sex sex as including “oral (or, for men only, anal) sex or any other contact involving the genital area” (Macdowall et al., 2013).

### Inclusion Criteria

Analysis was restricted to sexually experienced participants aged 17 to 24 at interview to ensure that the results were applicable to the contemporary population of young people who will have become sexually active since the turn of the millennium, and who will have been subject to similar social and cultural norms relating to first sex. This age group is of public health relevance as those within it are likely to be engaging in transient sexual relationships and are at particularly high risk of negative sexual health outcomes, such as STIs (Public Health England, 2012; Sonnenberg et al., 2013). To make appropriate statistical adjustments in the regression analyses, participants aged 16 at interview were excluded because they could not be ascribed an educational level. For purposes of consistency, we excluded 16-year-olds from all analyses presented in this article.

**Figure 1.**
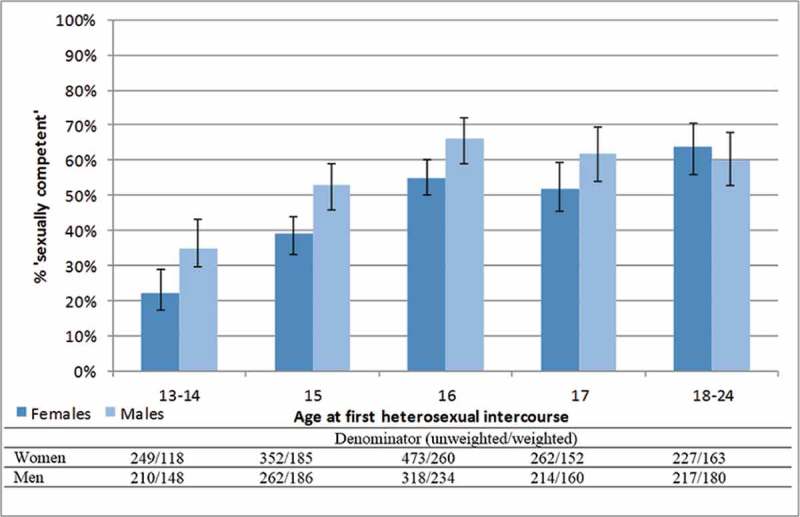
Percentage (95% CI) of sexually active 17-24 year olds^a^ who were 'sexually competent' at first intercourse, by age at first intercourse. ^a^Denominator is participants aged 17-24 at interview, who reported first heterosexual intercourse aged 13+. Participants reporting that they were ‘forced’ at first intercourse excluded (n=22).

The restriction of the sample to participants aged 17 to 24 who had ever had heterosexual intercourse will mean that, overall, there is an overrepresentation of participants who have had sex at younger ages, which is associated with being categorised as not sexually competent (see **Figure 1**) and other indicators of poor sexual health. This should be noted when interpreting the prevalence of key variables among this sample. A sensitivity analysis was conducted whereby the regression analyses were repeated among those aged 18 to 24 at interview (by 18, about 80% of participants reported experience of heterosexual intercourse; results shown in Supplementary Material).

Participants reporting that their partner was “more willing” at first sex were presented an additional question asking whether they were “forced.” Those who reported that their first sexual intercourse was forced were excluded from all analyses (*n* = 22; 5.5% of participants who reported that their partner had been “more willing”), as it was felt that it would be inappropriate to classify these participants in terms of sexual competence at first intercourse.

Specific restrictions to the sample were also made for analyses involving particular outcomes. Where the experience of nonvolitional sex was the outcome of interest, those who reported that the last occasion of nonvolitional sex occurred at an age earlier than their reported consensual sexual debut were excluded from analyses, in order that the sexual encounter at which sexual competence was or was not demonstrated preceded the occasion of nonvolitional sex. In analyses using unplanned pregnancy as the outcome, the sample was restricted so that only pregnancies with known outcomes were included, in other words, those pregnant at interview were excluded to avoid overrepresenting pregnancies resulting in birth, which are more likely to be planned. The corresponding number of participants excluded is stated in the footnotes of Table 2.

### Statistical Analysis

We graphically present the proportion of young men and women who were sexually competent at first intercourse, by age at which first intercourse occurred. We also calculate the prevalence of each outcome variable in the sexually active sample of 17- to 24-year-olds under focus in this study.

We assessed the associations between sexual competence at first sex, and sex before 16, and each indicator of subsequent sexual health using univariate logistic regression. Multivariable logistic regression was employed to determine whether sexual competence at first sex, and sex before 16, were independently associated with subsequent sexual health after adjustment for potential confounders. In the multivariable analysis we ran two models for each sexual health outcome. The first model was mutually adjusted for sexual competence at first sex and sex before 16. In addition, the second model also adjusted for area-level deprivation (measured using the Index of Multiple Deprivation; Payne & Abel, 2012), educational level, family structure at age 14 (whether lived with both natural parents “more or less continuously” until age 14), ethnicity, ease discussing sexual matters with parent(s) at age 14, main source of sex education, and duration sexually active.

The aim of the multivariable logistic regression was not to build the most parsimonious predictive model for each of the outcomes under study but to examine whether sexual competence at first sex was associated with the selected sexual health outcomes, independently of whether sexual debut occurred before the age of 16 and other potential confounding factors. The selection of covariates included in the final multivariable models was informed by prior analyses identifying the predictors of sexual competence at sexual debut (Palmer, 2015; Palmer, Clarke, & Wellings, 2015; Wellings et al., 2001) and the sexual health literature. Indicators of individual and familial socioeconomic status (SES) have previously been shown to be associated with timing (Wellings et al., 2001) and condom use (Henderson et al., 2002) at first sex. Certain ethnic groups have been found to be less likely to use contraception at sexual debut (Coleman & Testa, 2007) and more likely to report pressure from their partner at first sex (Wight et al., 2008). Disrupted family structure has been found to be associated with earlier sexual debut (Lenciauskiene & Zaborskis, 2008; Wellings et al., 2001) and lower reported “wantedness” of first sex (Abma, Driscoll, & Moore, 1998). School as the main source of sex education has been associated with contraceptive use and later sexual debut (Macdowall et al., 2015; Wellings et al., 2001).

Analyses involving the outcomes of STIs and low sexual function were carried out separately for men and women to allow for identification of gender differences. Unplanned pregnancy and nonvolitional sex were analyzed only among the female sample (a previous study found the prevalence of nonvolitional sex among 16- to 24-year-old men to be too low, at 0.8%, to permit these analyses; Macdowall et al., 2013). All statistical analyses were conducted using the Stata (Version 13) survey commands to account for the weighting, clustering, and stratification of the survey data. The HPV urine test data had specific weights applied that corrected for unequal probabilities of urine sample collection and differential sample response (Erens et al., 2013).

## Results

In the sample of sexually experienced 17- to 24-year-olds, 51.7% (95% CI: 48.9, 54.5) of women and 43.6% (95% CI: 40.4, 46.9) of men were classified as not sexually competent at first sex. No significant gender difference was observed in the proportion reporting heterosexual intercourse before age 16: 34.5% (95% CI: 31.9, 37.2) of women and 36.7% (95% CI: 33.7, 39.7) of men. Figure 1 presents the percentage of participants who were sexually competent at first intercourse, by age at first intercourse. Though the proportion classified as sexually competent increases with age at first sex (*p* < 0.05), age at first sex does not explain all of the variation observed in sexual competence. The equivalent analyses for each component of the sexual competence measure are available elsewhere (Palmer, 2015; Palmer, Clarke, & Wellings, 2015).

Table 2 presents the proportions of sexually active 17- to 24-year-olds reporting/experiencing key sexual health indicators, by gender. Significant gender differences were observed in the proportion who reported ever being diagnosed with an STI. Furthermore, HPV was detected in the urine of almost half of women and less than one-fifth of men; this difference is likely because urine is a less sensitive specimen type for men (Bissett et al., 2011). Table 3 presents the results of the regression analyses examining whether lack of sexual competence at first sex, and earlier first intercourse, predict subsequent sexual health among female participants. Having not been sexually competent at first sex was associated with increased odds of ever having had an STI diagnosis, testing positive for HPV at interview, experiencing low sexual function and having had an unplanned pregnancy (both in the year prior to interview), and experience of nonvolitional sex. These associations were statistically significant in the unadjusted analysis and remained significant when adjusting for multiple explanatory variables (see footnotes of Tables 3 and 4). First intercourse before 16 was associated with self-reported STIs, testing positive for HPV, unplanned pregnancy, and nonvolitional sex in the crude analyses, though no significant association was observed with low sexual function. When adjusted for multiple explanatory variables, including duration sexually active (see footnotes of Tables 3 and 4), sex before 16 retained a statistically significant association only with unplanned pregnancy and nonvolitional sex.

**Table 3.** Percentage (95% CI) of sexually active 17-24s who report/experienced outcomes of interest by sexual competence at first intercourse and reported sex before 16. Results of logistic regression analyses assessing association with: reported diagnosis of an STI ever, testing positive for HPV at interview, low sexual function in year prior to interview, unplanned pregnancy in year prior to interview, and reporting non-volitional sex ever (Women)

	Percentage with outcome (95% CI)	N (unweighted/ weighted)	Crude OR	95% CI	p-value	AOR1	95% CI	p-value	AOR2	95% CI	p-value
SELF-REPORTED STI (EVER)											
Sexual competence at first intercourse											
Sexually competent	14.9 (12.2, 18.1)	729/ 421	1			1			1		
Not sexually competent	24.4 (21.1, 28.1)	820/450	1.83	1.35,2.48	<0.001	1.64	1.19,2.25	0.002	1.46	1.06,2.02	0.022
Age at first intercourse											
≥16	16.2 (13.4, 19.4)	956/570	1			1			1		
<16	26.7 (23.1, 30.6)	598/302	1.86	1.38,2.51	<0.001	1.66	1.22,2.27	0.001	1.03	0.73,1.46	0.861
URINE-TEST HPV POSITIVE											
Sexual competence at first intercourse											
Sexually competent	40.2 (34.5,46.0)	383/237	1			1			1		
Not sexually competent	50.8 (45.8, 55.8)	476/281	1.53	1.11,2.10	0.010	1.44	1.04,1.99	0.029	1.56	1.11,2.18	0.010
Age at first intercourse											
≥16	42.3 (37.5, 47.3)	507/338	1			1			1		
<16	52.9 (47.3, 58.5)	353/181	1.52	1.11,2.06	0.008	1.42	1.04,1.94	0.028	1.17	0.83,1.65	0.363
LOW SEXUAL FUNCTION											
Sexual competence at first intercourse											
Sexually competent	9.1 (6.9, 11.9)	714/409	1			1			1		
Not sexually competent	17.3 (14.4, 20.5)	795/435	2.04	1.41,2.96	<0.001	2.03	1.38,2.99	<0.001	2	1.33,3.01	0.001
Age at first intercourse											
≥16	12.5 (10.2, 15.3)	927/550	1			1			1		
<16	14.9 (11.9, 18.3)	587/297	1.21	0.85,1.72	0.290	1.02	0.71,1.48	0.897	1.04	0.69,1.57	0.845
UNPLANNED PREGNANCY											
Sexual competence at first intercourse											
Sexually competent	1.6 (0.9, 2.8)	709/412	1			1			1		
Not sexually competent	4.1 (2.80, 5.9)	771/423	2.73	1.32,5.62	0.007	2.17	1.02,4.58	0.043	2.13	0.99,4.61	0.054
Age at first intercourse											
≥16	1.6 (0.9, 2.7)	923/552	1			1			1		
<16	5.2 (3.6, 7.6)	562/286	3.24	1.63,6.47	0.001	2.74	1.34,5.59	0.006	2.75	1.25,6.03	0.012
NON-VOLITIONAL SEX											
Sexual competence at first intercourse											
Sexually competent	3.3 (2.1, 5.1)	708/410	1			1			1		
Not sexually competent	10.6 (8.5, 13.2)	780/427	3.81	2.22,6.55	<0.001	2.98	1.70,5.23	<0.001	2.96	1.68,5.22	<0.001
Age at first intercourse											
≥16	3.8 (2.6, 5.5)	925/552	1			1			1		
<16	13.1 (10.4, 16.4)	568/287	3.88	2.41,6.24	<0.001	3.12	1.90,5.11	<0.001	3.13	1.87,5.23	<0.001

AOR1: Mutually adjusted for sexual competence and sex before 16. AOR2: Mutually adjusted for sexual competence and sex before 16, and: IMD quintile of residence at interview, educational level of respondent, family structure at age 14, ethnicity, ease discussing sexual matters with their parent(s) at age 14, their main source of sex education, duration sexually active.

**Table 4.** Percentage (95% CI) of sexually active 17-24s who report/experienced outcomes of interest by sexual competence at first intercourse and reported sex before 16. Results of logistic regression analyses assessing association with: reported diagnosis of an STI ever, testing positive for HPV at interview, and low sexual function in year prior to interview (Men)

MEN											
	Percentage with outcome (95% CI)	N (unweighted/ weighted)	Crude OR	95% CI	p-value	AOR1	95% CI	p-value	AOR2	95% CI	p-value
SELF-REPORTED STI (EVER)											
Sexual competence at first intercourse											
Sexually competent	7.6 (5.6, 10.3)	693/510	1			1			1		
Not sexually competent	10.5 (7.9, 13.8)	519/391	1.38	0.86,2.21	0.186	1.19	0.75,1.89	0.460	1.14	0.71,1.84	0.591
Age at first intercourse											
≥16	6.4 (4.7, 8.6)	749/573	1			1			1		
<16	13.4 (10.2, 17.4)	468/330	2.45	1.55,3.89	<0.001	2.38	1.52,3.73	<0.001	1.27	0.76,2.12	0.365
URINE-TEST HPV POSITIVE											
Sexual competence at first intercourse											
Sexually competent	13.1 (10.0, 17.1)	390/284	1			1			1		
Not sexually competent	21.1 (16.3, 26.9)	315/243	1.76	1.11,2.79	0.017	1.61	1.00,2.61	0.050	1.82	1.08,3.07	0.026
Age at first intercourse											
≥16	12.5 (9.3, 16.5)	412/329	1			1			1		
<16	23.6 (18.4, 29.7)	297/201	2.01	1.25,3.23	0.004	1.88	1.16,3.05	0.011	1.15	0.70,1.89	0.591
LOW SEXUAL FUNCTION											
Sexual competence at first intercourse											
Sexually competent	12.1 (9.6,15.2)	662/488	1			1			1		
Not sexually competent	17.1 (13.7,21.0)	495/373	1.45	0.99,2.11	0.055	1.48	1.01,2.17	0.044	1.52	1.04,2.22	0.031
Age at first intercourse											
≥16	14.6 (11.9,17.7)	707/545	1			1			1		
<16	14.2 (11.1,18.0)	455/319	0.91	0.63,1.33	0.635	0.86	0.58,1.26	0.429	0.84	0.55,1.28	0.419

AOR1: Mutually adjusted for sexual competence and sex before 16. AOR2: Mutually adjusted for sexual competence and sex before 16, and: IMD quintile of residence at interview, educational level of respondent, family structure at age 14, ethnicity, ease discussing sexual matters with their parent(s) at age 14, their main source of sex education, duration sexually active.

Table 4 shows that, among men, having been not sexually competent at first sex was associated with an increased risk of testing positive for HPV at interview and experiencing low sexual function in the year prior to interview in the crude and adjusted regression analyses. No statistically significant association was observed between a lack of sexual competence at first intercourse and self-reported STI diagnosis. Sexual debut before 16 was found to be associated with self-reported STI diagnosis and testing positive for HPV in the crude analyses, though these associations became nonsignificant in the fully adjusted model.

The restriction of the sample to participants aged 17 to 24 at interview does not seem to bias the associations identified, with very similar results found in the supplementary analysis conducted among 18- to 24-year-olds (see Supplementary Material).

## Discussion

A substantial proportion of young people in Britain lack sexual competence at first intercourse. The prevalence of sexual competence peaks at about 60% among those who were aged 18 to 24 at sexual debut, suggesting there is much room for improvement in optimizing young people’s first sexual intercourse. Although correlated, age at first sex does not explain all variation observed in sexual competence, indicating that an exclusive focus on age at occurrence fails to capture the variation in the experiences of first intercourse that exist. Furthermore, this study finds that a lack of sexual competence at sexual debut is associated with a range of indicators of subsequent sexual health status, independently of age at sexual debut. Among women, lack of sexual competence was associated with reporting of previous STI diagnosis/es, testing positive for HPV, experiencing low sexual function, having unplanned pregnancies, and experiencing nonvolitional sex, even when adjusting for multiple potential confounders. Among men, lack of sexual competence was independently associated with having tested positive for HPV and experiencing low sexual function.

Compared with the measure of sexual competence, first intercourse before 16 was associated with fewer indicators of sexual health in the crude analyses: reporting STI diagnosis/es, testing positive for HPV, unplanned pregnancy, and the experience of nonvolitional sex. However, when adjusting for a range of potential confounders, sex before 16 was no longer significantly associated with self-reported STIs or testing positive for HPV in either gender. When adding the covariates to the model one by one (analysis not shown), it became apparent that this loss of association was due to the duration sexually active variable, suggesting that the association between age at sexual debut and STIs was because those who had sex before 16 have been sexually active longer and therefore have had a greater chance of exposure to STIs.

Several studies using data from the second and third Natsal surveys have included the current measure of sexual competence in their analyses. Mercer et al. (2005) showed lack of sexual competence at first sex to be associated with reporting sexual function problems, as did Mitchell et al. (2013) in age-adjusted analyses of Natsal-3 data. Also in accordance with the current study, Wellings et al. (2013) found that a lack of lack of sexual competence at first sex was associated with having had an unplanned pregnancy in the last year in their age-adjusted analyses.

The current findings are also consistent with research conducted in North America concerned with contextual and psychosocial aspects of first sexual intercourse and how these relate to subsequent sexual health. A negative experience of first sex has been shown to be associated with a range of health outcomes, including sexual dysfunction, sexual guilt, poorer general health, a higher likelihood of STIs, lower life satisfaction, and negative feelings about current sexual encounters (Else-Quest et al., 2005; Reissing et al., 2012; Smith & Shaffer, 2013). Furthermore, positive associations between protective contraceptive behaviors at first intercourse and subsequent sexual encounters have also been identified in previous studies (Shafii, Stovel, Davis, & Holmes, 2004; Shafii et al., 2007; Stulhofer et al., 2010).

It has been suggested that the context and experience of sexual debut influences subsequent sexual behavior through the establishment and maintenance of sexual scripts, schemas, or habits. Researchers emphasize the role of social learning, placing individuals on certain trajectories informed by their prior sexual experiences (Else-Quest et al., 2005; Morgan & Zurbriggen, 2007; Reissing et al., 2012; Smith & Shaffer, 2013). Shafii et al. (2004) also suggest the role of habit formation in the link between condom use at sexual debut and condom use at subsequent encounters. Indicators of recent condom use and number of sexual partners were not adjusted for in the current analyses given their likely position on the causal pathway; however, further analysis (not shown) provided evidence for an indirect effect of sexual competence on STIs/HPV through these variables.

Sexual behavior is influenced by a huge number of factors at the societal, familial, and individual level, with no single element accounting for much of the large variation observed. Thus, it seems unlikely that young people have a blank sexual canvas before their first sexual intercourse which dictates their sexual trajectory from then on. However, the nature of the first sexual experience is likely to be one of the many influences at work in contributing to the patterning of subsequent experience.

### Limitations

Several limitations must be noted when interpreting the results of the current study. The response rate to Natsal-3, at 57.7% (Erens et al., 2013), potentially limits representativeness of the findings. However, this rate of response is similar to that of other major social surveys undertaken at the same time as Natsal-3 (Craig & Mindell, 2011; Park, Clery, Curtice, Phillips, & Utting, 2012). Any inference of causality must be considered with caution given the cross-sectional nature of the study. Furthermore, it is possible that unknown and/or unmeasured confounders could account for the observed associations between sexual competence at sexual debut and indicators of sexual health. For example, individual characteristics such as self-efficacy, sensation seeking, and impulsivity were not measured in the Natsal survey but have previously been found to be associated with early sexual intercourse and risky sexual behavior (Baele, Dusseldorp, & Maes, 2001; Castro, Bermudez, Buela-Casal, & Madrid, 2011; Charnigo et al., 2012; Hoyle, Fejfar, & Miller, 2000; Wulfert & Wan, 1993). The survey also lacked measures of other potential explanatory factors relating to sexual experiences that occurred prior to first intercourse, such as childhood sexual abuse (Leonard & Follette, 2002; Meston, Rellini, & Heiman, 2006) and consensual noncoital sexual practices (Halpern-Felsher et al., 2005; Lewis et al., 2013; O’Sullivan & Brooks-Gunn, 2005). Furthermore, though we are able to speculate about potential pathways of effect through the influence of social learning and the establishment of sexual scripts, we are unable to test these hypotheses, as no such information relating to these factors was collected in Natsal-3.

Reliance on retrospective reporting in the Natsal studies also has the potential to introduce recall bias. In restricting the age range of participants included in the current study to 17- to 24-year-olds, the time between the occurrence of first sexual intercourse and being interviewed will have been minimized to an extent—though this restriction is likely to have introduced bias into our prevalence estimates through the overrepresentation of people who started having sex at younger ages. The interpretation of events that take place early in life is likely to be shaped by subsequent experience and so cannot be construed simply as rationalization of past events (Giddens, 1992). This is not necessarily an inherent weakness of the study or the measure of sexual competence; we are not concerned with describing the experience as a fixed and observable reality but in terms of the way in which men and women construct and reflect on their first sexual experiences. Nevertheless, the quality of one’s current sex life is likely to color recollections of past sexual experiences, whether in an unfavorable or favorable light, and could introduce bias in the observed associations between the nature of first sex and subsequent sexual health status.

The measure of sexual competence itself was constructed somewhat opportunistically, using existing questions in the Natsal questionnaire, rather than inductively developing a conceptual model informed by qualitative research into young peoples’ experience of first sex (Wellings et al., 2001). This may have resulted in the omission of important components, such as experience of enjoyment, which, due to the reported gender differences in the priorities for and experiences of first sex, may be of particular importance for the way in which young men reflect on their first intercourse (Carpenter, 2002; DeLamater, 1986; Petersen & Hyde, 2011). However, despite its relatively crude beginnings, the finding that this measure of first intercourse is predictive of subsequent sexual health provides some empirical justification for the way in which it has been constructed.

When interpreting these findings we must note that, in answering these survey questions, respondents can consider the event only as they experienced it themselves, as one-half of the dyad. Consensuality within a sexual interaction must be two-sided, though sexual intent is often interpreted and established through body language and ambiguous communication (Mitchell & Wellings, 2002). Previous research suggests that men are more likely than women to perceive sexual intent (Farris, Treat, Viken, & McFall, 2008); and compared with women, young men more commonly report equal willingness of both partners at first intercourse (Wellings et al., 2001). This gendered discrepancy in the proportion reporting equal willingness might indicate that a male participant’s report of equal willingness at first sex may not reflect the encounter as experienced by his partner.

This measure accounts for only first *intercourse* as opposed to other first sexual activities. However, given that penetrative intercourse is commonly regarded as what constitutes “proper sex” by young heterosexuals, (Holland et al., 2000/2010), it is possible that specifically first intercourse is the most salient “first” in shaping subsequent sexual trajectories. It also should be noted that the questions used to construct the measure of sexual competence were asked only about first *heterosexual* sex, and so we cannot comment on the relevance of these results to young people whose first sexual experiences were with same-sex partners.

The outcome measure of HPV has its own limitations. For one, the sensitivity of the urine test for HPV is far lower among men than women (Bissett et al., 2011), meaning that the true prevalence among sexually active 17- to 24-year-old men is likely higher than that detected in the Natsal-3 survey, and this misclassification error should be noted when interpreting the results. Furthermore, given the rather high prevalence of HPV (low-risk and high-risk types) (Sonnenberg et al., 2013), it is questionable whether this measure should be considered as an indicator of poor sexual health. However, the measure was included in these analyses to explore whether the associations identified with the outcome of self-reported STIs were reflected in a biological measure of STIs. Furthermore, previous research has shown HPV infection to be associated with other indicators of sexual risk, including multiple sexual partners, concurrency, nonuse of condoms, and infection with other STIs (Burchell, Winer, de Sanjosé, & Franco, 2006).

### Conclusions and Implications

In contrast to the prevailing focus on the factors associated with “early” sexual debut, an increasing body of research has investigated the importance of the circumstances of, and feelings about, first sex and their potential role in shaping the subsequent sexual trajectory (Reissing et al., 2012; Smith & Shaffer, 2013). This study contributes to this emerging literature and provides evidence for the utility of a measure of sexual competence at sexual debut in identifying those at greater risk of poor sexual health. Sexual competence at first intercourse was found to be associated with subsequent sexual health status independently of, and in some cases more strongly than, age at sexual debut. This was particularly the case among women, for whom sexual competence was predictive of all five sexual health outcomes investigated.

Our unadjusted analyses found sex before age 16 to be associated with self-reported STIs and testing positive for HPV among both genders, and unplanned pregnancy and nonvolitional sex among women. Therefore, in terms of identifying individuals at heightened risk of poor sexual health, the more conventionally and easily measured age at first sex remains useful—though it shows no association with low sexual functioning. However, in conceptualizing the experience in a more nuanced way by using this measure of sexual competence, our hope is that we have offered a more meaningful approach to assessing the appropriateness of timing of sexual debut in a public health context.

Our findings have important implications for public health practice. Much sexual behavior–oriented intervention research, particularly that concerned with sex and relationship education, has focused on delaying sexual activity in terms of chronological age, though evidence for the effectiveness of these efforts is mixed (Kirby, 2001; Kirby, Laris, & Rolleri, 2007; Macdowall et al., 2015; Wight et al., 2002). Given previous qualitative research suggesting young people see the legitimacy of engaging in sex better located in the notion of “readiness,” as opposed to age-defined legality (Thomson, 2004), sexual competence at first sex may represent an alternative goal for interventions that is more agreeable to young people themselves. In shifting the focus from a problematization of adolescent sexual intercourse to a more accepting approach concerned with transitioning into sexual activity in a healthy and positive manner defined by circumstance rather than age, such efforts may be more compatible with the priorities of the young people targeted. If we accept that optimizing the experience of first sex in itself is a worthwhile goal, then the chance that these efforts may also translate into better subsequent sexual health serves to strengthen the argument for a shift in the educational and research paradigm concerned with young persons’ sexual behavior and health.

U.K. government guidance states that sex and relationship education (SRE) should enable young people to protect their physical sexual health and also equip them to handle the relational and emotional aspects of sexual activity, with reference to negotiation skills and resisting pressure (Department for Education and Employment, 2000). It is in relation to the latter in particular that school provision of SRE is lacking (Office for Standards in Education Children’s Services and Skills, Ofsted, 2010), despite young people themselves reporting a desire for a stronger focus on the relational aspects of sexual activity (Macdowall et al., 2006; Tanton et al., 2015).

The components that make up the measure of sexual competence are reflected in educational and advisory materials aimed at young people, which encourage consideration of the ideal circumstances and conditions for first having sex, as opposed to the right age at which to do so. The U.K. government Teenage Pregnancy Strategy media campaign “Sex: Worth Talking About,” for example, features a webpage titled “Are you ready for sex?” (National Health Service, 2009) that advises young people to consider whether they feel pressure from their partner or are trying to impress their friends, how they might feel about it afterward, and whether they are equipped with reliable contraception. This is a good example of how considerations relating to the circumstances and context of first sexual intercourse can be used practically to support young people in their decision to become sexually active. It is hoped that our finding of an association between sexual competence and subsequent sexual health goes some way toward providing the empirical basis for, and so ratifying, an emphasis on comprehensive sex education that provides young people with the skills required to embark on sexual activity which is physically, emotionally, and socially healthy.

## Supplementary Material

Supplementary Tables
