# Supplementary material for: Is “Sexual Competence” at First Heterosexual Intercourse Associated With Subsequent Sexual Health Status?
Source: J Sex Res. 2016 Feb 18;54(1):91–104. doi: 10.1080/00224499.2015.1134424 (PMC5214675; doi:10.1080/00224499.2015.1134424)
Supplement: Supplementary Tables [file hjsr_a_1134424_sm8626.doc]

SUPPLEMENTARY TABLE (1): Percentage (95% CI) of sexually active 18-24s who report/experienced outcomes of interest by sexual competence at first intercourse and reported sex before 16. Results of logistic regression analyses assessing association with: reported diagnosis of an STI ever, testing positive for HPV at interview, low sexual function in year prior to interview, unplanned pregnancy in year prior to interview, and reporting non-volitional sex ever (Women)

| WOMEN (18-24) | Percentage with outcome (95% CI) | N (unweighted/ weighted) | Crude OR | 95% CI | p-value | AOR1 | 95% CI | p-value | AOR2 | 95% CI | p-value |
| --- | --- | --- | --- | --- | --- | --- | --- | --- | --- | --- | --- |
| **SELF-REPORTED STI (EVER)** |  |  |  |  |  |  |  |  |  |  |  |
| **Sexual competence at first intercourse** |  |  |  |  |  |  |  |  |  |  |  |
| Sexually competent | 16.0 (13.0, 19.4) | 661/387.89 |  |  |  |  |  |  |  |  |  |
| Not sexually competent | 26.6 (23.0, 30.6) | 725/403.78 | 1.77 | 1.29,2.42 | <0.001 | 1.61 | 1.17,2.23 | 0.004 | 1.46 | 1.05,2.02 | 0.024 |
| **Age at first intercourse** |  |  |  |  |  |  |  |  |  |  |  |
| ≥16 | 17.2 (14.2, 20.6) | 867/526.69 |  |  |  |  |  |  |  |  |  |
| <16 | 29.7 (25.7, 34.0) | 524/267.45 | 1.74 | 1.28,2.39 | 0.001 | 1.58 | 1.15,2.18 | 0.005 | 1.16 | 0.81,1.68 | 0.411 |
| **URINE-TEST HPV POSITIVE** |  |  |  |  |  |  |  |  |  |  |  |
| **Sexual competence at first intercourse** |  |  |  |  |  |  |  |  |  |  |  |
| Sexually competent | 40.4 (34.5, 46.6) | 344/219.09 |  |  |  |  |  |  |  |  |  |
| Not sexually competent | 50.6 (45.3, 56.0) | 416/250.94 | 1.49 | 1.06,2.10 | 0.021 | 1.4 | 0.99,1.99 | 0.056 | 1.48 | 1.03,2.13 | 0.032 |
| **Age at first intercourse** |  |  |  |  |  |  |  |  |  |  |  |
| ≥16 | 42.1 (37.1, 47.3) | 458/313.61 |  |  |  |  |  |  |  |  |  |
| <16 | 53.5 (47.5, 59.5) | 303/156.92 | 1.56 | 1.13,2.16 | 0.007 | 1.46 | 1.05,2.03 | 0.023 | 1.18 | 0.81,1.70 | 0.386 |
| **LOW SEXUAL FUNCTION** |  |  |  |  |  |  |  |  |  |  |  |
| **Sexual competence at first intercourse** |  |  |  |  |  |  |  |  |  |  |  |
| Sexually competent | 9.7 (7.3, 12.7) | 646/376.87 |  |  |  |  |  |  |  |  |  |
| Not sexually competent | 17.8 (14.7, 21.3) | 703/390.46 | 1.97 | 1.34,2.88 | 0.001 | 1.96 | 1.32,2.93 | 0.001 | 1.96 | 1.28,3.00 | 0.002 |
| **Age at first intercourse** |  |  |  |  |  |  |  |  |  |  |  |
| ≥16 | 13.1 (10.7, 16.1) | 838/506.03 |  |  |  |  |  |  |  |  |  |
| <16 | 15.2 (12.1, 19.0) | 516/263.77 | 1.17 | 0.81,1.70 | 0.396 | 1.01 | 0.68,1.48 | 0.978 | 1.05 | 0.68,1.62 | 0.817 |
| **UNPLANNED PREGNANCY** |  |  |  |  |  |  |  |  |  |  |  |
| **Sexual competence at first intercourse** |  |  |  |  |  |  |  |  |  |  |  |
| Sexually competent | 1.6 (0.9, 2.9) | 640/378.39 |  |  |  |  |  |  |  |  |  |
| Not sexually competent | 3.9 (2.6, 5.8) | 680/379.26 | 2.65 | 1.24,5.68 | 0.012 | 2.15 | 0.97,4.75 | 0.059 | 2.18 | 0.95,4.99 | 0.065 |
| **Age at first intercourse** |  |  |  |  |  |  |  |  |  |  |  |
| ≥16 | 1.6 (0.9, 2.9) | 833/507.49 |  |  |  |  |  |  |  |  |  |
| <16 | 4.9 (3.3, 7.3) | 492/252.64 | 3.05 | 1.48,6.30 | 0.003 | 2.59 | 1.22,5.52 | 0.013 | 2.76 | 1.21,6.32 | 0.016 |
| **NON-VOLITIONAL SEX** |  |  |  |  |  |  |  |  |  |  |  |
| **Sexual competence at first intercourse** |  |  |  |  |  |  |  |  |  |  |  |
| Sexually competent | 3.4 (2.1, 5.3) | 639/376.7 |  |  |  |  |  |  |  |  |  |
| Not sexually competent | 10.7 (8.5, 13.5) | 691/383.17 | 3.73 | 2.13,6.53 | <0.001 | 2.96 | 1.65,5.33 | <0.001 | 2.91 | 1.61,5.26 | <0.001 |
| **Age at first intercourse** |  |  |  |  |  |  |  |  |  |  |  |
| ≥16 | 4.0 (2.7, 5.8) | 837/508.91 |  |  |  |  |  |  |  |  |  |
| <16 | 13.2 (10.3, 16.8) | 498/253.43 | 3.68 | 2.25,6.04 | <0.001 | 2.98 | 1.77,5.00 | <0.001 | 3.06 | 1.79,5.22 | <0.001 |

SUPPLEMENTARY TABLE (2): Percentage (95% CI) of sexually active 17-24s who report/experienced outcomes of interest by sexual competence at first intercourse and reported sex before 16. Results of logistic regression analyses assessing association with: reported diagnosis of an STI ever, testing positive for HPV at interview, and low sexual function in year prior to interview (Men)

| MEN (18-24) | Percentage with outcome (95% CI) | N (unweighted/ weighted) | Crude OR | 95% CI | p-value | AOR1 | 95% CI | p-value | AOR2 | 95% CI | p-value |
| --- | --- | --- | --- | --- | --- | --- | --- | --- | --- | --- | --- |
| **SELF-REPORTED STI (EVER)** |  |  |  |  |  |  |  |  |  |  |  |
| **Sexual competence at first intercourse** |  |  |  |  |  |  |  |  |  |  |  |
| Sexually competent | 8.1 (6.0, 11.0) | 620/466.83 |  |  |  |  |  |  |  |  |  |
| Not sexually competent | 11.0 (8.2, 14.6) | 466/360.24 | 1.32 | 0.81,2.15 | 0.260 | 1.14 | 0.71,1.83 | 0.588 | 1.11 | 0.69,1.81 | 0.662 |
| **Age at first intercourse** |  |  |  |  |  |  |  |  |  |  |  |
| ≥16 | 6.5 (4.8, 8.8) | 694/540.39 |  |  |  |  |  |  |  |  |  |
| <16 | 15.1 (11.5, 19.5) | 395/288.78 | 2.41 | 1.50,3.88 | <0.001 | 2.36 | 1.48,3.75 | <0.001 | 1.38 | 0.81,2.34 | 0.234 |
| **URINE-TEST HPV POSITIVE** |  |  |  |  |  |  |  |  |  |  |  |
| **Sexual competence at first intercourse** |  |  |  |  |  |  |  |  |  |  |  |
| Sexually competent | 13.6 (10.2, 17.8) | 346/258.28 |  |  |  |  |  |  |  |  |  |
| Not sexually competent | 21.8 (16.7, 27.9) | 284/225.65 | 1.78 | 1.10,2.89 | 0.019 | 1.63 | 0.99,2.69 | 0.054 | 1.84 | 1.07,3.18 | 0.029 |
| **Age at first intercourse** |  |  |  |  |  |  |  |  |  |  |  |
| ≥16 | 13.2 (9.8, 17.5) | 379/308.73 |  |  |  |  |  |  |  |  |  |
| <16 | 24.5 (18.9, 31.1) | 253/177.44 | 1.99 | 1.22,3.24 | 0.006 | 1.85 | 1.12,3.04 | 0.016 | 1.13 | 0.67,1.92 | 0.647 |
| **LOW SEXUAL FUNCTION** |  |  |  |  |  |  |  |  |  |  |  |
| **Sexual competence at first intercourse** |  |  |  |  |  |  |  |  |  |  |  |
| Sexually competent | 12.4 (9.7, 15.6) | 589/444.32 |  |  |  |  |  |  |  |  |  |
| Not sexually competent | 17.0(13.5, 21.1) | 445/343.97 | 1.4 | 0.94,2.07 | 0.094 | 1.45 | 0.97,2.16 | 0.071 | 1.48 | 0.99,2.22 | 0.054 |
| **Age at first intercourse** |  |  |  |  |  |  |  |  |  |  |  |
| ≥16 | 14.9 (12.2, 18.2) | 653/512.1 |  |  |  |  |  |  |  |  |  |
| <16 | 13.9 (10.6, 18.1) | 384/278.3 | 0.85 | 0.56,1.29 | 0.445 | 0.8 | 0.52,1.22 | 0.302 | 0.75 | 0.46,1.21 | 0.235 |

AOR1: Mutually adjusted for sexual competence and sex before 16

AOR2: Mutually adjusted for sexual competence and sex before 16, and: IMD quintile of residence at interview, educational level of respondent, family structure at age 14, ethnicity, ease discussing sexual matters with their parent(s) at age 14, their main source of sex education, duration sexually active
